# Intelligence, &c.

**Published:** 1824-09-01

**Authors:** 


### INTELLIGENCE, &c.

☞ *The following was transmitted to us by the Two-penny Post.*

### SOCIETY OF PHYSICIANS of the UNITED KINGDOM.

ESTABLISHED IN LONDON, JUNE 17, 1824.

ALTHOUGH Medicine has been studied from a very early period, and considerable genius and learning have been employed in its cultivation, yet such is the extreme complication and difficulty of the subject, that its present state still admits of great improvement; to which, perhaps, nothing would more effectually contribute, than the intimate union and active cooperation of its professors.

Much may certainly be accomplished by united effort, which individual exertion, however well directed, is unable to effect. While, at the same time, it cannot be doubted, that whatever contributes to the advancement of Medical Science, must, by increasing its usefulness, add to the dignity of the profession.

Under these impressions, and considering that a great majority of the regular Graduates in Physic, of this Country, are at present in an isolated state; several Physicians practising in London, have been induced to associate; and to invite the zealous co-operation throughout the Kingdom, of that part of the Profession to which they belong; with a confident hope of facilitating, by these means, the accomplishment of the laudable purposes just mentioned.

It is, therefore, proposed that a Society be established, having principally in view the following objects:—

I. The reception and discussion of subjects connected, in any manner, with the science of Medicine.

II. The combined investigation of such points, whether theoretical or practical, as are at present obscure or uncertain, and to the elucidation of which, individual labour has hitherto appeared inadequate.

III. The publication of papers furnished by Members of the Society, or of those which may be transmitted to them, by the Profession at large.

IV. And, in general, the effecting of whatever may tend to improve the science of Medicine, or to advance the interests and dignity of its Professors, the regularly educated Graduates in Physic of the Universities of the United Kingdom.

*At a Meeting held JUNE 17th, 1824, at the House of Dr. SHEARMAN,*

Present;—Drs. TEMPLE, CLEVERLY, BIRKBECK, UWINS, CLUTTERBUCK, HANCOCK, SHEARMAN, COPLAND, TWEEDIE, and ROBERTS.

*It was resolved unanimously*—

I. That a Society of Physicians be established, for the purposes above stated.

II. That it be called "*The Society of Physicians of the United Kingdom.*"

III. That the Society consist of such persons only, as have actually prosecuted the study of Medicine in a University, for the period prescribed by its regulations, and who, having subsequently submitted to the usual tests and examinations, have thereby obtained the degree of Bachelor or Doctor of Physic. *But Members of the London College, whether Fellows or Licentiates, admitted prior to the year* 1800, *are eligible.*

IV. *That no person be a member of this Society, who is engaged in the actual practice of Surgery, Pharmacy, or Midwifery.*

V. That a Committee be appointed for the purpose of giving the necessary publicity to these transactions; of receiving communications from the Profession; of preparing a system of laws and regulations, for the government of the Society; and of performing, in general, whatever may be conducive to its interests, prior to the first General Meeting; to which they are to report proceedings, and resign their functions.

VI. That the following Gentlemen be Members of this Committee, with the power of making such additions to their number, as they may judge convenient:—

*Committee:*—Drs. TEMPLE, CLEVERLY, BIRKBECK, UWINS, CLUTTERBUCK.

VII. That the first General Meeting take place at the House of Dr. BIRKBECK, at half-past Eight in the Evening, on the second Thursday in October next.

(SIGNED) C. J. ROBERTS, *Sec. pro temp.*

Communications on the subject of the Society, to be addressed to Dr. ROBERTS, No 20, Earl Street, Blackfriars.

### REMARKS.

We have but a very few observations to make on the laws of this Society, which, from its organization, must quickly fall into decay, or even annihilation. *If the objects of the Society were what they seem to be*—the improvement of Medical Science—the MEANS would not be restricted to such narrow channels as in the above code. Are Surgery, Pharmacy, and Midwifery no parts of Medical Science?* Can this extensive and most difficult of all branches of human knowledge be cultivated and improved only by men who have graduated at particular Universities? Is the man who has spent twenty, thirty, or forty years, in the ardent pursuit of medical knowledge, in various countries, *necessarily* incapable of promoting medical Science, if he have not spent two years in Glasgow, or three in Edinburgh?—Is it in the 19th Century that such illiberal principles are to be publicly avowed by a Society, the private and well-known object of which is, to oppose similar, but far more liberal restrictions, enjoined by the Royal College of Physicians? Is it in the 19th Century that such a principle is maintained, as that, *the man who is admitted a Licentiate of the Royal College of Physicians, on an examination only*, in 1799, is capable of advancing the interests of the Medical Profession, while *all others*, admitted in a similar manner, after that period, are totally incapable, and

* Has the principal Member of this Junto forgot that he himself practised very many years as an Apothecary, and, in *such capacity*, considered himself qualified to be a Censor of Medical Science, having published no less than 15 Volumes of a MEDICAL REVIEW? Is the ladder by which he ascended to be kicked away the moment he arrives at the summit?

consequently unfit to be members of this medical Junto? Such is one of the fundamental laws of the Society—a law obviously made, not from any zeal for science, but to include one or two personal friends of the Junto, and to exclude all others. Finally, we do suspect that the *ostensible* are not the real objects of this society—that it is erected on the basis of injustice and illiberality—and that it will end in discomfiture and contempt.

---

II.

*Artificial Seltzer Water.* This celebrated Mineral Water is now imitated, with great accuracy, at Brighton, and sent to all parts of the kingdom. An establishment, under the direction of Dr. Struve, of Dresden, has been formed on the Marine Parade, and from personal experience, we can vouch for the luxury, as well as salubrity of this excellent beverage: It is more grateful than Soda Water, while it improves the state of the digestive organs, and, through them, gives tone to the whole system.

---

III.

*Literary Intelligence.* The Papers printed in the Transactions of the Royal Society, during the last three years, detailing the Discoveries of the Functions of the Nerves, will be immediately re-published with Notes, and a general introductory View of the Nervous System; by Mr. Charles Bell, Professor of Anatomy and Surgery to the Royal College of Surgeons, and Surgeon to the Middlesex Hospital.

---

IV.

*Protection of Vaccination.* Dr. Gregory, the able Physician to the Small-Pox Hospital, has just published his Report to the Governors of that Institution. We can only notice those particulars that relate to Vaccination. In 1823, of 151 patients, treated in the Institution for Small Pox, 47 had previously undergone vaccination. *The whole of these recovered.* On a medium calculation, twelve of these would have died had it not been for the protecting influence of vaccination—though there is reason to believe, that it was but imperfectly performed in these 47 cases. Of the 72 admitted in the first five months of 1824, nineteen had been vaccinated. Of these, one died. In this unfortunate case, Dr. Gregory ascertained that the vaccine process had been very imperfect. In 1823, there were vaccinated, under Dr. Gregory's superintendance, 3129 persons, and in the first five months of 1824, one thousand two hundred and eighty passed through this process. Dr. G. avers that, at no previous period, has the confidence of the lower orders in the security of vaccination appeared greater than at present. The metropolitan population, Dr. G. observes, have better opportunities of appreciating the protecting influence of vaccination than those of the country towns, as small pox is always present in London—"seeking whom it may devour." Dr. G. concludes with a high, and well-merited eulogium on the Jennerian discovery.

---

TO THE FACULTY.

A Surgeon who has been established in a large and populous Town and Neighbourhood, for upwards of Four and Twenty Years, as a general Practitioner, is anxious to dispose of it immediately.

The Terms will be found highly advantageous to the Purchaser. Reference is requested to be made to Dr. James Johnson, of *Suffolk Place, Pall Mall East, London,* who will give every information on the subject, that may be required.

